# Evaluation of public health surveillance systems in refugee settlements in Uganda, 2016–2019: lessons learned

**DOI:** 10.1186/s13031-022-00449-x

**Published:** 2022-04-08

**Authors:** Alex Riolexus Ario, Emily Atuheire Barigye, Innocent Harbert Nkonwa, Jimmy Ogwal, Denis Nixon Opio, Lilian Bulage, Daniel Kadobera, Paul Edward Okello, Leocadia Warren Kwagonza, Susan Kizito, Benon Kwesiga, Julius Kasozi

**Affiliations:** 1 Ministry of Health of Uganda, Kampala, Uganda; 2 Uganda Public Health Fellowship Program, Ministry of Health, Kampala, Uganda; 3 Uganda National Institute of Public Health, Kampala, Uganda; 4 African Field Epidemiology Network, Kampala, Uganda; 5 United Nations High Commissioner for Refugees, Kampala, Uganda

**Keywords:** Public health surveillance, Refugee, Learned, Uganda

## Abstract

**Background:**

Civil wars in the Great Lakes region resulted in massive displacement of people to neighboring countries including Uganda. With associated disease epidemics related to this conflict, a disease surveillance system was established aiming for timely detection of diseases and rapid response to outbreaks. We describe the evaluation of and lessons learned from the public health surveillance system set up in refugee settlements in Uganda.

**Methods:**

We conducted a cross-sectional survey using the US Centers for Disease Control and Prevention Updated Guidelines for Evaluating Public Health Surveillance Systems and the Uganda National Technical Guidelines for Integrated Disease Surveillance and Response in four refugee settlements in Uganda—Bidibidi, Adjumani, Kiryandongo and Rhino Camp. Using semi-structured questionnaires, key informant and focus group discussion guides, we interviewed 53 health facility leaders, 12 key personnel and 224 village health team members from 53 health facilities and 112 villages and assessed key surveillance functions and attributes.

**Results:**

All health facilities assessed had key surveillance staff; 60% were trained on Integrated Disease Surveillance and Response and most village health teams were trained on disease surveillance. Case detection was at 55%; facilities lacked standard case definitions and were using parallel Implementing Partner driven reporting systems. Recording was at 79% and reporting was at 81%. Data analysis and interpretation was at 49%. Confirmation of outbreaks and events was at 76%. Preparedness was at 72%. Response was at 34%. Feedback was at 82%. Evaluate and improve the system was at 67%. There was low capacity for detection, response and data analysis and interpretation of cases (< 60%).

**Conclusion:**

The surveillance system in the refugee settlements was functional with many performing attributes but with many remaining gaps. There was low capacity for detection, response and data analysis and interpretation in all the refugee settlements. There is need for improvement to align surveillance systems in refugee settlements with the mainstream surveillance system in the country. Implementing Partners should be urged to offer support for surveillance and training of surveillance staff on Integrated Disease Surveillance and Response to maintain effective surveillance functions. Functionalization of district teams ensures achievement of surveillance functions and attributes. Regular supervision of and support to health facility surveillance personnel is essential. Harmonization of reporting improves surveillance functions and attributes and appropriation of funds by government to districts to support refugee settlements is complementary to maintain effective surveillance of priority diseases in the northern and central part of Uganda.

## Introduction

**Fig. 1:**
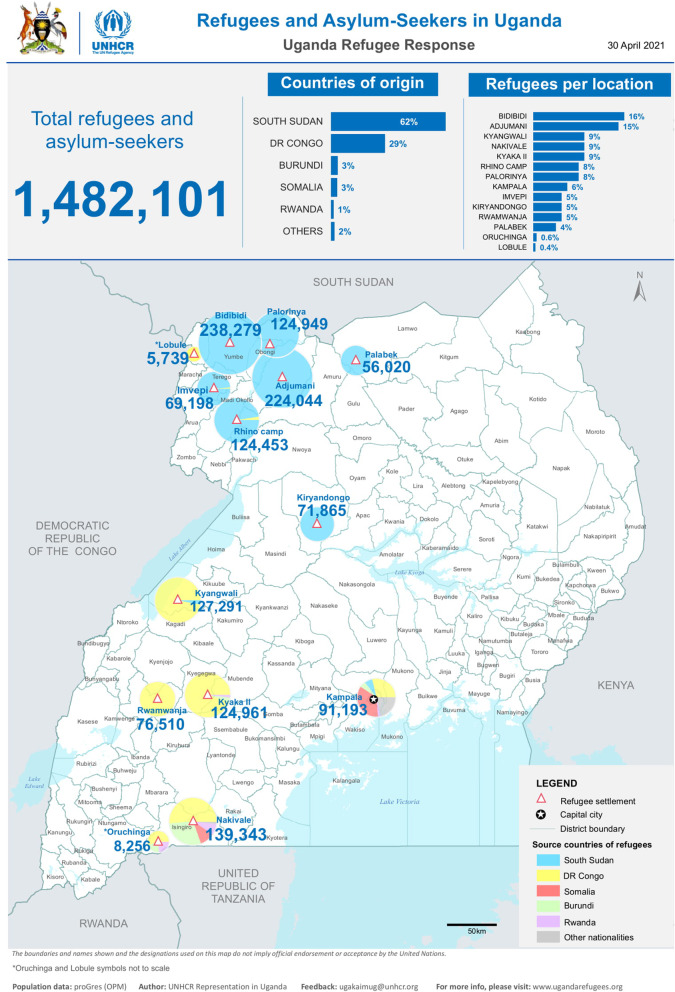
Location of refugee settlements in Uganda, 2021. NB: Adapted from UNHCR archives

The civil wars and fragile states in the Great Lakes region of Eastern Africa has resulted in massive displacement of people to neighboring countries including Uganda whose open refugee policy has contributed to its bigger refugee population [1]. South Sudan, which gained independence on July 9, 2011, had an outbreak of an inter-ethnic war which accounts for the majority of refugees in Uganda [2]. The refugee settlements in Uganda, as shown in Fig. 1, include: Adjumani, Bidibidi, Imvepi, Kampala, Kiryandongo, Kyaka II, Kyangwali, Lobule, Nakivale, Oruchinga, Palabek, Palorinya, and Rwamwanja [3].

The total population in these settlements was 1,434,708 by October, 2020; this number continues to increase with renewed conflict and hostilities that have plagued the region [4]. People are often displaced as a result of humanitarian crises. Those affected are settled in locations with high population densities, inadequate food and shelter, unsafe water, poor sanitation, and lack of infrastructure. These circumstances increase risk of transmission of communicable diseases and acute malnutrition with its antecedent consequences of increased morbidity and mortality [5]. Reliable and appropriate disease surveillance enables timely detection and response to disease outbreaks and public health challenges.

Uganda’s Ministry of Health (MoH) has implemented the Integrated Disease Surveillance and Response (IDSR) as a national strategy for improving epidemiologic surveillance and response since 2000 [6]. The strategy, which was developed by the World Health Organization (WHO) Regional Office for Africa, provides a framework for implementing the requirements of the International Health Regulations (IHR) at the country level. The Integrated Disease Surveillance and Response (IDSR) approach aims at coordinating and integrating surveillance activities including: detection, confirmation/verification, notification/reporting; and timely response to control and prevention of disease outbreaks and other public health events of national and international concern. The IDSR system is supposed to be used in all health systems including refugee settlements in the country. During humanitarian emergencies, public health surveillance systems tend to be disrupted and/or unable to adequately address surveillance functions [7]. Evaluation of a public health surveillance system provides evidence-based information, which could be used for strengthening the reporting mechanism and enhancing implementation of public health actions [8]. We describe the evaluation of the public health surveillance system in refugee settlements in Uganda in which the surveillance functions and surveillance attributes were assessed and lessons learned documented.

## Methods

### Study design

We conducted a cross-sectional survey using the US Centers for Disease Control and Prevention (CDC) Updated Guidelines for Evaluating Public Health Surveillance Systems [9] and the Uganda National Technical Guidelines for Integrated Disease Surveillance and Response (IDSR) [6].

### Study site

The study sites were the refugee settlements of Bidibidi, Adjumani, Kiryandongo and Rhino Camp located in Northern and Central Uganda. Bidibidi Refugee Settlement covers an area of 250 sq. km and is located in Yumbe District, Northern Uganda. The settlement had a capacity of 180,000, but there were 238,279 refugees mainly from South Sudan. Adjumani Refugee Settlement comprises 17 camps, and is located in Adjumani District. The total refugee population for Adjumani District stood at 224,044. Kiryandongo Refugee Settlement, located in Kiryandongo District, had a population of 71,865. Rhino Camp Refugee Settlement located in Arua District, and spread over 3 sub-counties (Rigbo, Odupi and Uriama), had a population of 124,453.

### Sampling and sample size

Using simple random sampling, we picked 6 health facilities in Adjumani, 16 in Bidibidi, 24 in Kiryandongo and 7 in Rhino Camp. Sample size for the clusters (villages) was calculated using sample size calculator software (vSphere) to pick clusters [10]. Inter-cluster correlation was estimated to be 0.3 and a baseline compliance rate was assumed to be 50%. Accordingly, 24 villages were selected from Bidibidi and Kiryandongo, and 32 villages were selected from Adjumani and Rhino Camp Refugee Settlements. From each village, 2 Village Health Team members were selected for interviews on a first found first picked basis.

### Data collection

We conducted observations and interviews (Table 1). We interviewed 53 health facility leaders and 53 surveillance focal persons, 4 chairmen of the District Epidemic Preparedness and Response Committees and 224 Village Health Team members using a semi-structured questionnaire.

**Table 1:** Definition, data sources and method of collection of surveillance functions and attributes

Attribute/function	Definition	Data source(s)	Method of data collection
Simplicity	Structure and ease of operation	Health facility staff	Interview using semi-structured questionnaire
Flexibility	Ability to adapt to changing operating conditions with little additional cost in time, personnel or funds	Registers	Observation
Data quality	A measure of how well suited a dataset serves its intended purpose	Registers	Observation
Acceptability	Willingness of individuals and organisations to participate in the surveillance system	Health facility staff	Interview using semi-structured questionnaire
Sensitivity	Case reporting—the proportion of cases of a disease detected by the surveillance system	Registers and health facility staff	Observation and interview using semi-structured questionnaire
Predictive value positive	The proportion of persons identified as cases who actually do have the condition under surveillance	Registers	Counts
Representative	Comparison of characteristics of reported events to actual events	Health facility staff	Observation
Timeliness	Speed or delay between steps in a surveillance system	Health facility staff	Interview using semi-structured questionnaire
Stability	Ability of a surveillance system to collect, manage and provide data without failure	Registers	Observation
Detection	Process of identifying presence of a disease or an event	Registers and health facility staff	Observation and interview using semi-structured questionnaire
Recording	Documenting information for future reference	Registers and health facility staff	Observation and interview using semi-structured questionnaire
Reporting	Written account of an event which has been observed or investigated	Health facility staff	Interview using semi-structured questionnaire
Availability of guidelines and tools	Process of assigning meaning to the collected information and determining the conclusions, significance and implications of the findings	Health facility	Observation
Training on IDSR	Laboratory diagnosis based on cases definition and set criteria in the national guidelines	Health facility staff	Interview using semi-structured questionnaire
Data analysis	A set of measures undertaken by an organization to better respond and cope with the aftermath of an outbreak or event of importance	Health facility	Observation and interview using semi-structured questionnaire
Confirmation of outbreaks	Measures which attempt to minimize the spread of or effects of a disease outbreak or an event	Registers and health facility staff	Observation and interview using semi-structured questionnaire
Preparedness	Information sent back to a health worker or responder in regard to a report or message earlier submitted	Health facility staff	Interview using semi-structured questionnaire
Response	Reassessing the surveillance functions and making recommendations for improving the quality and efficiency of the system	Health facility staff	Interview using semi-structured questionnaire
Feedback		Health facility staff	Interview using semi-structured questionnaire
Evaluate and improve system		Health facility staff	Interview using semi-structured questionnaire

Data was collected electronically using an Open Data Kit Software (Kobo Collect for Humanitarian Emergencies) using Tablet PCs.

The following surveillance functions were assessed—detection, recording, reporting, preparedness, response, feedback, confirmation of outbreaks, data analysis and interpretation. The surveillance attributes assessed are defined in Table 1. All the surveillance attributes were assessed since the system in the refugee settlement is supposed to be the same with all the other systems in the country.

**Fig. 2:**
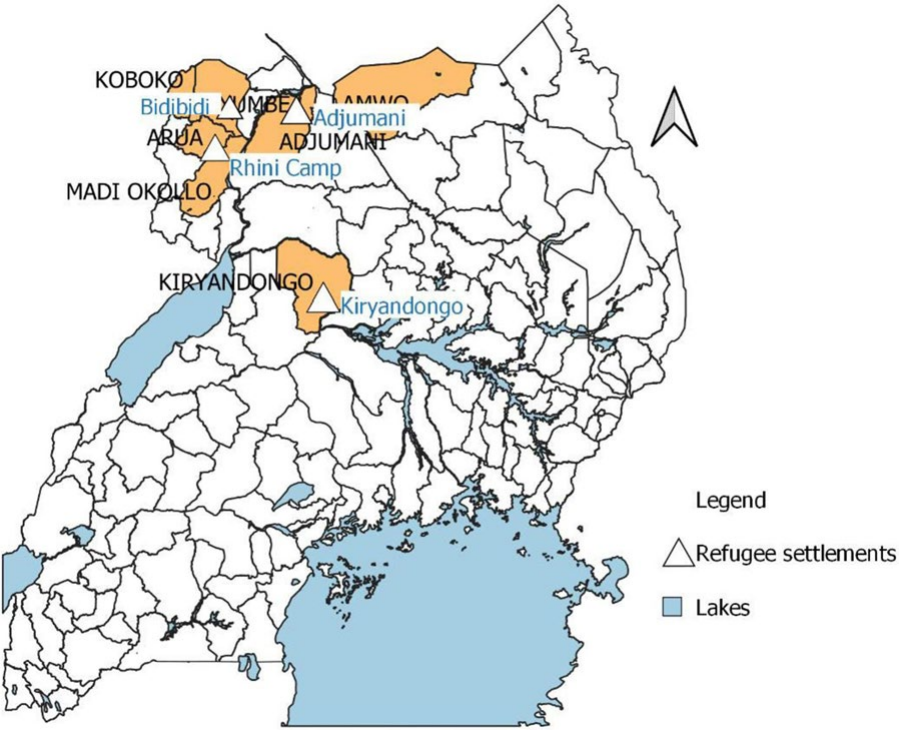
Map showing Adjumani, Bidibidi, Kiryandongo and Rhino Camp study sites

The capacity of the refugee settlements in performing surveillance functions (Table 2) and all the attributes (Table 3) of a public health surveillance system were assessed in the four refugee settlements of Adjumani, Bidibidi, Kiryandongo and Rhino Camp (Fig. 2) using health facility registers and reporting tools, semi-structured questionnaires, key informant interviews and focus group discussions.

**Table 2:** Capacity of refugee settlements in performing surveillance functions

Surveillance function	Average % score by refugee settlement			
	Bidibidi	Rhino Camp	Adjumani	Kiryandongo
Detection	55	56	53	57
Recording	77	90	83	67
Reporting	75	95	85	67
Data analysis and interpretation	19	95	50	33
Confirmation of outbreaks and events	50	85	86	83
Preparedness	72	68	65	83
Response	11	25	50	50
Feedback	75	75	79	100
Evaluate and improve system	55	70	60	83

**Table 3:** Surveillance attributes of refugee settlements as per evaluation assessment

Attribute	Description of a surveillance attribute by Refugee Settlement			
	Bidibidi	Rhino Camp	Adjumani	Kiryandongo
Simplicity	Moderate*	Moderate	Moderate	Moderate
Flexibility	Low	Low	Low	Low
Data quality	Moderate	Moderate	Moderate	Moderate
Acceptability	Moderate	Moderate	Moderate	Moderate
Sensitivity	76%	77%	78%	83%
Predictive value positive	50%	70%	66%	70%
Representativeness	Low	Low	Low	Low
Timeliness	52%	78%	76%	79%
Stability	Low	Low	Low	Low

*The categories were: low (< 60%), moderate (60–74%), and high (≥ 75%)

### Data analysis

We analyzed qualitative data using the content analysis model. We sorted and coded the data into categories, in order to bring together related terms. We edited and summarized useful information and developed conclusions without changing the meaning of what the respondents said. We used Epi-data to capture quantitative data and exported to Stata version 16 for analysis. We used descriptive statistics to summarize variables in form of rates and proportions. We calculated the positive predictive value of the system using cholera data by comparing the proportion of persons identified as having cholera to those who actually had the condition under surveillance. We categorized some attributes on a scale if many parameters describing one attribute were measured. The categories were: low (< 60%), moderate (60–74%), and high (≥ 75%).

## Results

All the 53 health facilities assessed had key surveillance staff such as clinicians, nurses, records assistants, laboratory and environmental health personnel employed by government or implementing partners. However, only 60% of health workers were trained on Integrated Disease Surveillance and Response. The VHT structure was in place and they were trained on Community Based Disease and Events Surveillance. Eighty percent of the facilities lacked evidence that VHTs were reporting on Community Based Disease Surveillance activities.

## Capacity in performing surveillance functions

All 53 facilities had Out Patient Department (OPD) registers feeding into the weekly and monthly reports. However, there was lack of standard case definitions (SCDs) and use of parallel Implementing Partner driven Health Information System (HIS). Detection of cases was at 55% (Range = 53–57) while recording was at 79% (Range = 67–90), and reporting was at 81% (Range = 67–95). Analysis and interpretation of data was at 49% (Range = 19–95). The capacity of the settlements to confirm outbreaks and events was at 76% (Range = 50–86) and their level of preparedness was at 72% (Range = 65–83). The response rate was at 34% (Range = 11–50) while ability to provide feedback was at 82% (Range = 75–100). The capacity of the system to evaluate and improve the system was at 67% (Range = 55–83).

## Surveillance attributes

### Simplicity

**Fig. 3:**
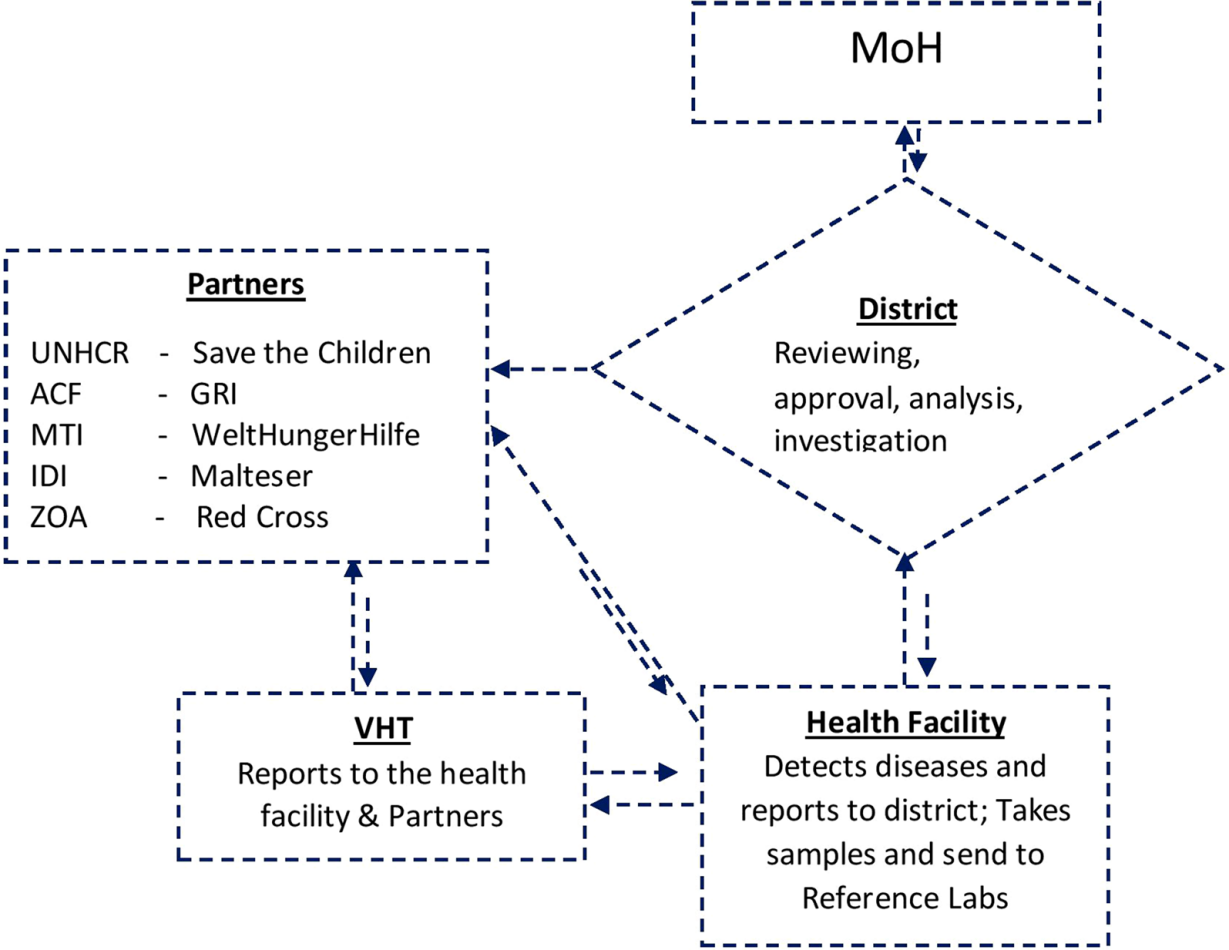
Data flow of the surveillance system in refugee settlements

Diagnoses and reporting of priority diseases were made according to standard case definitions in 65% (34/53) of the health facilities, 35% of the facilities had their own (IP driven) case definitions. No facility had drawn its own list of priority diseases, events or conditions, but used the list of diseases specified on the weekly reporting form “HMIS 033b” [10, 11]. Case based investigation forms were lacking in 65% of the health facilities. Simplified line lists for reporting common conditions such as measles were found with 55% of the VHTs. There were two reporting channels, one from VHTs and health facilities through the districts to the MoH and another to the Implementing Partner supporting a refugee settlement as shown in the flowchart in Fig. 3.

### Flexibility

There were two reporting systems with different reporting tools in health facilities. One reporting system was driven by the Implementing Partners while the other used the MoH system. Age categorization of sub-groups differed across the facilities from the standard MoH guidelines.

### Data quality

The registers had many gaps (e.g. key indicators such as suspected malaria, and next of kin were not there) and there were considerable missing data. Only 33% (17/53) of the health facilities assessed demonstrated the capacity to analyze and present data using charts and maps.

### Acceptability

The health workers, whether employed by government or partners, accepted that there were gaps in timeliness and completeness in reporting. Users interviewed expressed willingness to align the reporting system with the MoH standard reporting format.

### Sensitivity

The surveillance system was sensitive at 79% (Range = 76–83) in detecting suspected outbreaks during the period of study “April 2016 to December 2019”. Malaria cases reported in the study period were 105,159 in Kiryandongo, 144,873 in Adjumani, 137,424 in Rhino Camp, and 155,238 in Bidibidi, of which 428,728 tested positive by malaria rapid diagnostic test. The most reported epidemic prone diseases were: malaria, typhoid, dysentery, measles and cholera. However, only a few were investigated by the districts. Other events captured by the surveillance system were: animal bites (suspected Rabies), Adverse Events Following Immunization, presumptive multi-drug resistant tuberculosis, acute flaccid paralysis, malaria deaths, perinatal and maternal deaths.

### Positive predictive value

The positive predictive value for surveillance was 64% (Range = 50–70). There were 124 cases of cholera (40 in Kiryandongo, 12 in Rhino Camp, 6 in Bidibidi and 66 in Adjumani) reported of which 79 tested positive by culture at Uganda National Health Laboratory Services. Case investigations of cholera outbreaks were carried out during the study period by the response teams in Adjumani, Kiryandongo and Rhino Camp Refugee Settlements.

### Representativeness

Age categorization of sub-groups differed across the facilities from the standard MoH guidelines.

### Timeliness

The average weekly reporting rate in the refugee settlements was 71% (Range = 52–79). The district health teams reported inadequate skills to record, summarize and send reports on mTrac. mTrac is a mobile tracking system of the MoH where health workers send alert messages using phone numbers which have been entered into the District Health Information Software version 2. Few health workers were on the mTrac system. Outbreaks of cholera were reported within a week of occurrence in Adjumani, Kiryandongo and Rhino Camp Refugee Settlements.

### Stability

Internet connectivity challenges were experienced by all facilities visited and the District Health Office. Funds to repair equipment were in short supply in all four refugee settlements.

## Discussion

The surveillance system in the refugee settlements of Adjumani, Bidibidi, Kiryandongo and Rhino Camp demonstrated ability to detect and ensure that health conditions of public health importance are monitored efficiently and effectively to control and prevent epidemics. However, there were many gaps that could negatively affect the efficiency of the system. The system did not follow the MoH IDSR guidelines in its entirety. The core function of IDSR is to strengthen district level surveillance and response for priority diseases and integrate laboratory activities, reduce duplication in reporting, and share resources among disease control programs.

The surveillance functions of recording, reporting, preparedness, feedback and confirmation of outbreaks and events were highly achieved, and evaluate and improve the system was moderately achieved. However, there was low capacity for detection, response and data analysis and interpretation in all the refugee settlements. Lack of standard case definitions and use of parallel Implementing Partner driven Health Information System possibly contributed to this. In addition, most health workers were not trained on IDSR and hence lacked data quality and analytical skills. Drehobl et al. asserts that much as data systems that monitor health threats are becoming increasingly automated, human expertise is, and always will be, critical to recognizing potential cases of disease, diagnosing disease, reporting diseases or conditions, analyzing and interpreting data, and communicating results to all stakeholders [12]. For this reason, the nation’s health professionals from all disciplines and at all levels are fundamental to sustaining and enhancing public health surveillance capacity. Addressing skills and competency gap of the health professionals is a critical step in ensuring improved surveillance systems to effectively and efficiently perform surveillance functions. In the low scoring category in implementing surveillance functions, Bidibidi scored lowest compared to the other three refugee settlements. This could be attributed to Bidibidi being a very new settlement compared to the much older well-established settlements. Adokiya et al., assessed the core and support functions of the IDSR system at the periphery level of the health system in northern Ghana and made similar deductions on the gaps in the surveillance system. Their findings differed with ours on the feedback in that they found the feedback irregular in Ghana [13].

As shown in Table 3, sensitivity, timeliness and positive predictive value attributes scored highly in all refugee settlements except Bidibidi which scored relatively low in timeliness and positive predictive value. Simplicity, acceptability and data quality scored moderately while flexibility, representativeness and stability attributes were low in all the refugee settlements. Gazarian et al. assessed whether the national active surveillance of uncommon childhood conditions facilitated by the Australian Paediatric Surveillance Unit fulfilled its objectives, and found the positive predictive value to be 70% which is quite similar to our findings [14]. Hussain et all conducted an evaluation of acute respiratory infection surveillance systems in Gilgit-Baltistan, Pakistan, and found their system to be simple, with high sensitivity but less flexible and with moderate data quality [15]. This is quite similar to our findings.

## Conclusion

The surveillance system in the refugee settlements was functional with many performing attributes but with many remaining gaps. There was low capacity for detection, response and data analysis and interpretation of cases in all the refugee settlements. There is need for improvement to align surveillance systems in refugee settlements with the mainstream surveillance system in the country. Implementing Partners should be urged to offer support for surveillance and training of surveillance staff on Integrated Disease Surveillance and Response to maintain effective surveillance functions. Functionalization of district teams ensures achievement of surveillance functions and attributes. Regular supervision of and support to health facility surveillance personnel is essential. Harmonization of reporting improves surveillance functions and attributes and appropriation of funds by government to districts to support refugee settlements is complementary to maintain effective surveillance of priority diseases in the Northern and Central part of Uganda.

## Data Availability

Participant data without names and identifiers will be made available after approval from the corresponding author and Uganda National Institute of Public Health, Ministry of Health, Kampala, Uganda.

## Abbreviations

CBDS: Community Based Disease Surveillance
CDC: US Centers for Disease Control and Prevention
CIF: Case Investigation Form
DEPRC: District Epidemic Preparedness and Response Team
DHO: District Health Office
DHT: District Health Team
DRRT: District Rapid Response Team
HIS: Health Information System
HMIS: Health Management Information System
IDSR: Integrated Disease Surveillance and Response
IHR: International Health Regulations
IP: Implementing Partner
NRRT: National Rapid Response Team
MoH: Ministry of Health
SCD: Standard Case Definition
VHT: Village Health Team
UNHCR: United High Commissioner for Refugees
WHO: World Health Organisation
